# *Microcystis aeruginosa* strengthens the advantage of *Daphnia similoides* in competition with *Moina micrura*

**DOI:** 10.1038/s41598-017-10844-7

**Published:** 2017-08-31

**Authors:** Hengxing Tang, Xinying Hou, Xiaofeng Xue, Rui Chen, Xuexia Zhu, Yuan Huang, Yafen Chen

**Affiliations:** 1 Jiangsu Key Laboratory for Biodiversity and Biotechnology, School of Biological Sciences, Nanjing Normal University, 1 Wenyuan Road, Nanjing, 210046 China; 2 State Key Laboratory of Lake and Environment, Nanjing Institute of Geography and Limnology, Chinese Academy of Sciences, 73 East Beijing Road, Nanjing, 210008 China

## Abstract

*Microcystis* blooms are generally associated with zooplankton shifts by disturbing interspecific relationships. The influence of *Microcystis* on competitive dominance by different sized zooplanktons showed species-specific dependence. We evaluated the competitive responses of small *Moina micrura* and large *Daphnia similoides* to the presence of *Microcystis* using mixed diets comprising 0%, 20%, and 35% of toxic *M*. *aeruginosa*, and the rest of green alga *Chlorella pyrenoidosa*. No competitive exclusion occurred for the two species under the tested diet combinations. In the absence of *M*. *aeruginosa*, the biomasses of the two cladocerans were decreased by the competition between them. However, the *Daphnia* was less inhibited with the higher biomass, suggesting the competitive dominance of *Daphnia*. *M*. *aeruginosa* treatment suppressed the population growths of the two cladocerans, with the reduced carrying capacities. Nonetheless, the population inhibition of *Daphnia* by competition was alleviated by the increased *Microcystis* proportion in diet. As a result, the competitive advantage of *Daphnia* became more pronounced, as indicated by the higher *Daphnia*: *Moina* biomass ratio with increased *Microcystis* proportions. These results suggested that *M*. *aeruginosa* strengthens the advantage of *D*. *similoides* in competition with *M*. *micrura*, which contributes to the diversified zooplankton shifts observed in fields during cyanobacteria blooms.

## Introduction

Cyanobacteria blooms occur with increased frequency, persistence and wide water ranges due to the eutrophication associated with global warming^1^. These aggravated blooms often lead to adverse changes in aquatic ecosystem properties, including toxin production, weakened trophic cascades, and deterioration of water quality^2, 3^.

Compared with other phytoplankton, cyanobacteria are generally accepted as poor food reducing zooplankton fitness. The production of toxic metabolites including microcystins usually causes sublethal or lethal effects for zooplankton survival^4^. The deficiency in nutrition like sterols and long-chained polyunsaturated fatty acids suppresses the carbon metabolism and thereby declines the zooplankton growth^5, 6^. In addition, the colonial or filamentous morphology in cyanobacteria inhibits the grazing activity by clogging the zooplankton filtering apparatus^7, 8^. Nonetheless, in the context of “arms-race” hypothesis, the zooplankton develop adaptations to alleviate the harmful effects by cyanobacteria^9, 10^. For example, some copepods can avoid the ingestion of toxic cells via detecting cyanobacterial metabolites based on the selective feeding^11, 12^. A short-time previous exposure to cyanobacteria improves the fitness of some cladocerans, which could be transferred to offspring via maternal effects^13–15^. In addition, zooplankton can develop cyanobacteria-tolerant genotypes via rapid evolution^16^. These phenotypic and genotypic adaptions are thought to affect the species shifts and community structures of zooplankton during cyanobacteria blooms^17^.

Competition is one of the forces structuring zooplankton community. The competition between zooplankton in the presence of cyanobacteria has been widely studied. Most literatures stated that cyanobacteria support the competitive dominance from large sized species to small ones, e.g., from *Daphnia* to smaller cladocerans^18–21^. Nonetheless, some investigations demonstrated that copepods or large cladocerans are superior competitors in cyanobacterial environment^22–24^. Given these incompatible results in literatures and the zooplankton adaptions to cyanobacteria, the competition shift during blooms can be interpreted as the dominance by better adapted zooplankton species. As these adaptions are induced by exposure to cyanobacteria, it is hypothesized that the competitive advantage can be affected by varied cyanobacteria stress. To test the hypothesis, we co-cultivated the small-sized *Moina micrura* and large-sized *Daphnia similoides* by feeding diets comprising 0%, 20%, and 35% of toxic *M*. *aeruginosa*. The objective of the present study was to compare the competitions between the two cladocerans under different *Microcystis* stresses. As no competitive exclusion was observed during the cultivation, the species that has relatively higher biomass in competition was defined as the superior competitor.

## Results

### Population dynamics in monocultures

**Figure 1:**
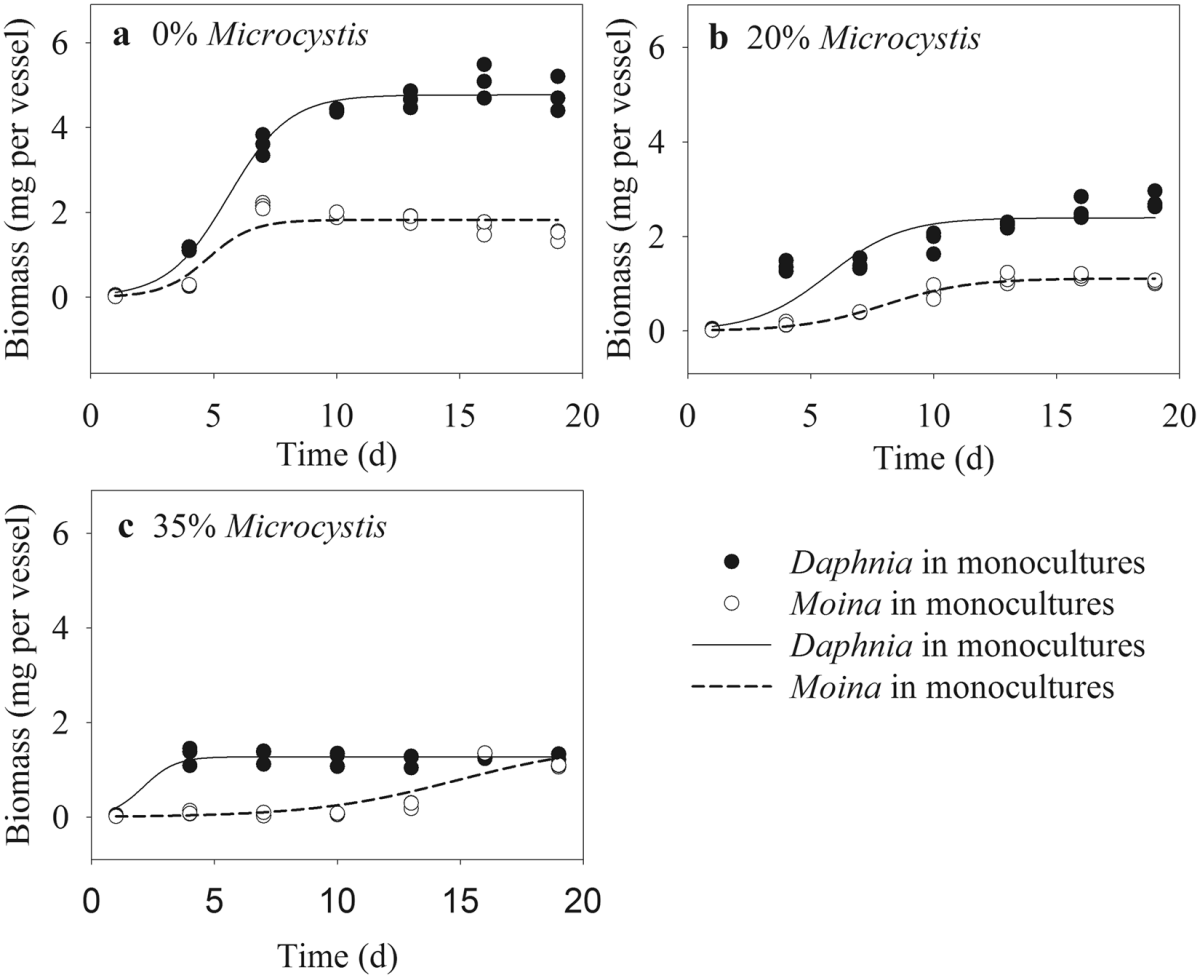
Population growth curves of *D*. *similoides* and *M*. *micrura* in monocultures with different *Microcystis* proportions in food. Both the two cladocerans have three replicates on each day. Some of the data points overlap because they have almost identical values. Lines represent non-line regression (Logistic model).

The biomasses of the two cladocerans generally increased with progressing culture time among all groups (Fig. 1). Nonetheless, the maximum biomass of both *Daphnia* and *Moina* decreased with increased *Microcystis* proportions in food. As the *Microcystis* proportion increased from 0% to 35%, the maximum biomass of cladocerans decreased from ~5.1 mg to ~1.4 mg per vessel for *Daphnia*, and decreased from ~2.2 mg to ~1.2 mg per vessel for *Moina*. *Microcystis* significantly affected the time reaching the maximum biomass (Fig. 1 and Table 1). Corresponding to the increased *Microcystis* proportion to 35% in food, the time reaching the maximum biomass was shortened from 16 days to 4 days for *Daphnia*, but was prolonged from 7 days to 16 days for *Moina* (Fig. 1).

**Table 1:** Results of two-way ANOVA on maximum biomass, time to maximum biomass, carrying capacity and population growth rate of *D*. *similoides* and *M*. *micrura* subjected to different food combinations and absence/presence of competitor (DF: degree of freedom; SS: sum of squares; MS: mean squares; F-F ratio).

Traits	*DF*	*SS*	*MS*	*F*	*P*
**Maximum biomass**					
*D*. *similoides*					
Food combination (A)	2	17.793	8.896	181.100	<0.001
Presence of competitor (B)	1	8.046	8.046	163.787	<0.001
A × B	2	5.783	2.891	58.857	<0.001
*M*. *micrura*					
Food combination (A)	2	1.704	0.852	218.665	<0.001
Presence of competitor (B)	1	3.000	3.000	769.836	<0.001
A × B	2	0.443	0.221	56.788	<0.001
**Time to maximum biomass**					
*D*. *similoides*					
Food combination (A)	2	351.000	175.500	10.324	0.002
Presence of competitor (B)	1	72.000	72.000	4.235	0.062
A × B	2	147.000	73.500	4.324	0.039
*M*. *micrura*					
Food combination (A)	2	325.000	162.500	108.333	<0.001
Presence of competitor (B)	1	0.500	0.500	0.333	0.574
A × B	2	7.000	3.500	2.333	0.139
**Carrying capacity (** ***K*** **)**					
*D*. *similoides*					
Food combination (A)	2	15.301	7.651	248.312	<0.001
Presence of competitor (B)	1	5.680	5.680	184.364	<0.001
A × B	2	5.527	2.763	89.693	<0.001
*M*. *micrura*					
Food combination (A)	2	0.674	0.337	41.486	<0.001
Presence of competitor (B)	1	3.248	3.248	399.832	<0.001
A × B	2	0.246	0.123	15.159	<0.001
**Population growth rate (** ***r*** **)**					
*D*. *similoides*					
Food combination (A)	2	1.431	0.716	12.685	<0.001
Presence of competitor (B)	1	0.148	0.148	2.624	0.131
A × B	2	0.590	0.295	5.230	0.023
*M*. *micrura*					
Food combination (A)	2	1.750	0.875	152.820	<0.001
Presence of competitor (B)	1	0.0105	0.0105	1.839	0.200
A × B	2	0.0151	0.00757	1.319	0.304

### Population dynamics in cocultures

**Figure 2:**
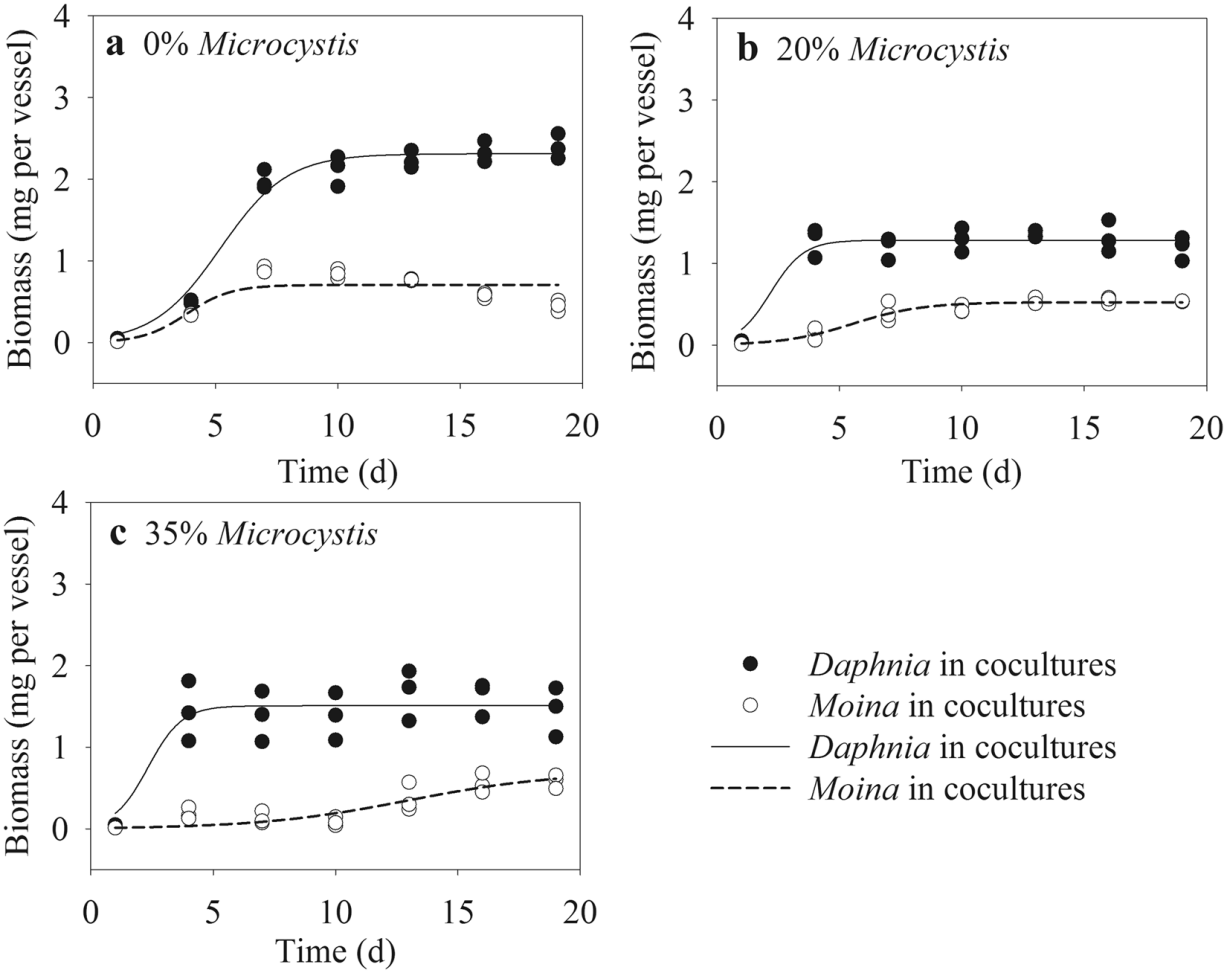
Population growth curves of *D*. *similoides* and *M*. *micrura* in cocultures with different *Microcystis* proportions in food. Both the two cladocerans have three replicates on each day. Some of the data points overlap because they have almost identical values. Lines represent non-line regression (Logistic model).

In general, *Daphnia* had higher biomasses than *Moina* did in all cocultures (Fig. 2). When fed 100% *Chlorella*, the biomass of *Daphnia* rapidly increased to ~2.4 mg per vessel on day 7, after which the biomass increased slightly. By contrast, the biomass of *Moina* gradually decreased from day 7 when a peak biomass of ~0.9 mg per vessel was reached. With 20% *Microcystis* in food, *Daphnia* reached its peak biomass of 1.43 mg per vessel on day 4. The biomass of *Moina* increased slowly with a maximum value of 0.56 mg per vessel on day 13. The population dynamics of *Daphnia* at 35% *Microcystis* was comparable to that at 20% *Microcystis*. By contrast, there was only minor increase in the biomass of *Moina* before day 13, and *Moina* reached its peak biomass of ~0.6 mg per vessel on day 16 at 35% *Microcystis* (Fig. 2).

### Biomass inhibition of species and biomass ratio in competition

**Figure 3:**
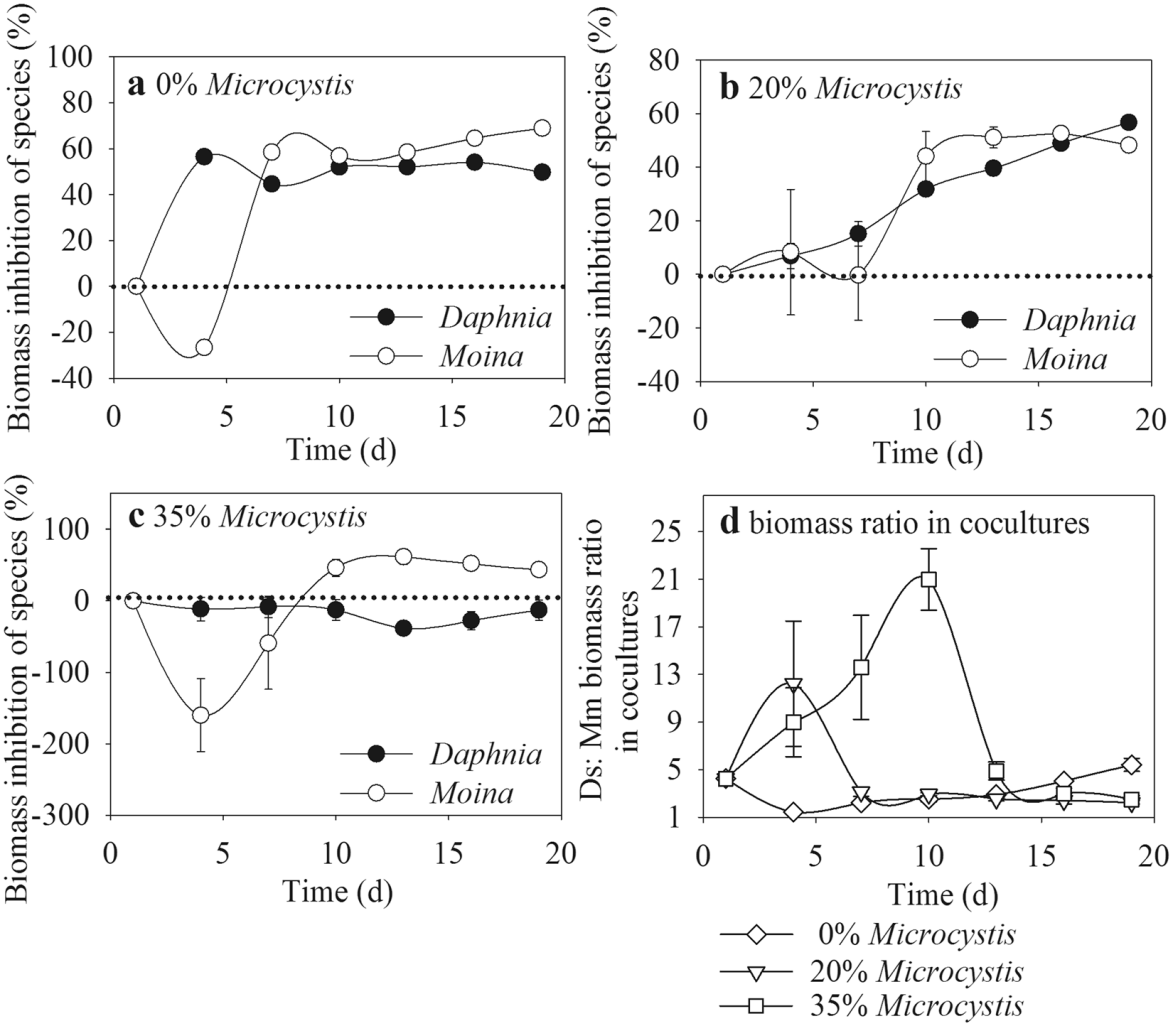
Biomass inhibition rates of species (**a**–**c**) and the biomass ratio between *D*. *similoides* (Ds) and *M*. *micrura* (Mm) in co-cultures (**d**) with different *Microcystis* proportions in food.

The biomass inhibition of the two species changed with time depending on the *Microcystis* proportion (Fig. 3a–c). Without *Microcystis* addition, the *Daphnia* biomass in competition was sharply inhibited by ~57% during the initial 3 days, whereas that of *Moina* was promoted by the presence of *Daphnia*, as indicated by the negative values of biomass inhibition rate. Nonetheless, the biomass inhibition of *Moina* dramatically increased to ~61%, and was higher than that of *Daphnia* after 7 days (Fig. 3a). With 20% *Microcystis* in food, although the biomass inhibition of *Daphnia* increased along with time, it reached a maximum value of ~59% at the end of experiment, which was rapidly achieved on day 3 in groups without *Microcystis*. The biomass inhibition rate of *Moina* varied around that of *Daphnia* (Fig. 3b). With 35% *Microcystis*, although negative values were observed for the biomass inhibition of *Moina* at the initial 7 days, it sharply increased to ~49% from day 9. Nonetheless, the biomass inhibition rate of *Daphnia* was negative and approached zero during the experiment period (Fig. 3c).

The *Daphnia*: *Moina* biomass ratio provided an intuitive understanding for the differences in population dynamics between the two cladocerans (Fig. 3d). In cocultures, the biomass of *Daphnia* was always higher than that of *Moina*, regardless of the *Microcystis* treatment, as indicated by the ratio values >1. There were remarkable increases in the biomass ratio with increased *Microcystis* proportions in food. Nonetheless, the peak value of the ratio at 35% *Microcystis* appeared later than that at 20% *Microcystis* did.

### Environmental carrying capacity and population growth rate

**Figure 4:**
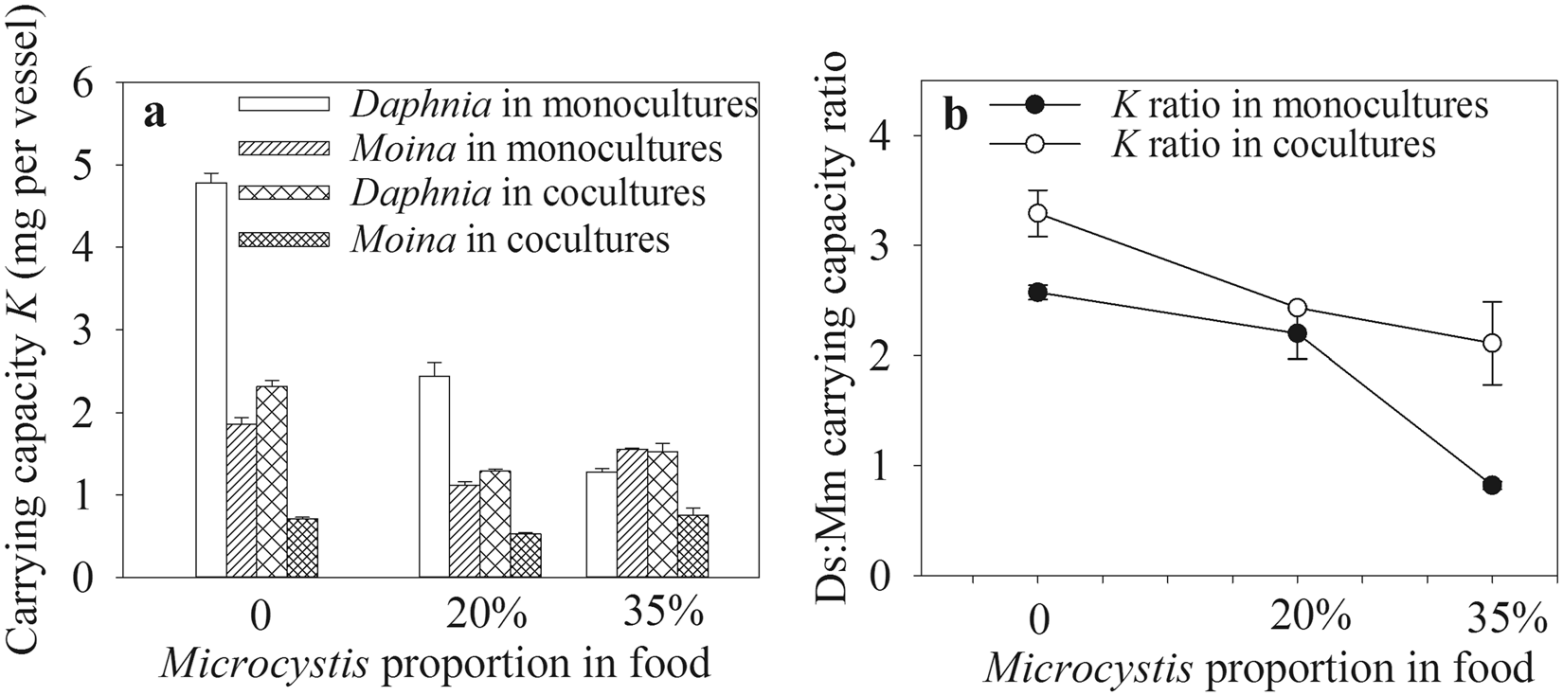
Carrying capacities (**a**) of *D*. *similoides* and *M*. *micrura* and their ratios in monocultures or cocultures (**b**) with different *Microcystis* proportions in food.

The carrying capacities of both *Daphnia* and *Moina* were significantly affected by the *Microcystis* treatment, the presence of competitor and the interactions between them (Table 1). When fed 100% *Chlorella*, the carrying capacities of *Daphnia* and *Moina* were decreased by 51.7% and 62.4% by the presence of each other in cocultures. This phenomenon was also observed in populations fed 20% *Microcystis*, together with the overall decreased carrying capacities. At 35% *Microcystis*, the carrying capacity of *Daphnia* in cocultures was higher by 21.4% than that in monocultures, whereas the carrying capacity of *Moina* was decreased by 51.9% in competition (Fig. 4a). *Daphnia* had higher carrying capacities than the *Moina* did in all cultures except for the case in monocultures at 35% *Microcysits*. The *Daphnia*: *Moina* carrying capacity ratio in cocultures was always higher than that in monocultures, although decreasing trends were observed with increased *Microcystis* proportions in food (Fig. 4b).

**Figure 5:**
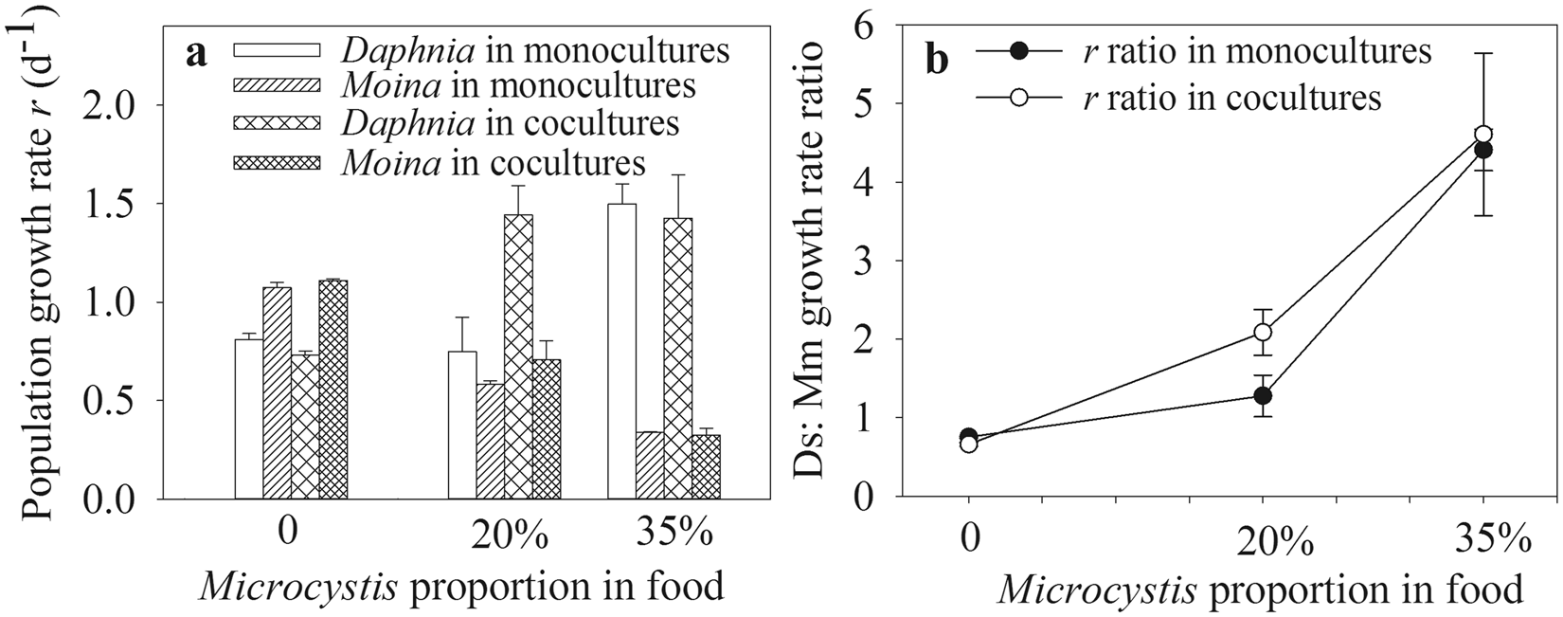
Population growth rates (**a**) of *D*. *similoides* and *M*. *micrura* and their ratios in monocultures or cocultures (**b**) with different *Microcystis* proportions in food.

The population growth rates (*r*) of the two cladocerans were significantly affected by the *Microcystis* treatment, but not the competition (Table 1). When fed 100% *Chlorella*, the *r* of *Moina* was higher by ~42% than that of *Daphnia*. *Microcystis* treatment increased the *r* of *Daphnia*, but decreased the *r* of *Moina*. The *r* of *Daphnia* was remarkable higher by 50.7% at 20% *Microcystis* and by 77.6% at 35% *Microcystis* than those of *Moina* in cocultures, leading to the increased *Daphnia*: *Moina* growth rate ratio with increased *Microcystis* proportions in food (Fig. 5).

## Discussion

The present study revealed that the large-sized *D*. *similoides* was superior to the small-sized *M*. *micrura* in competition under favorable food condition. When fed 100% *Chlorella*, *D*. *similoides* had higher biomass than the *M*. *micrura* did in cocultures, although the biomasses of the two cladocerans were suppressed by the competition (Figs 1 and 2). This is in accordance with the previous conclusion that abundant edible food favours the larger species to be superior competitor^25–27^. On condition that the carbon levels satisfy the food requirements of the animals, large species gathers food more efficiently, thereby decreasing the food availability of small species^28^. The large animals also generally have the stronger ability to ingest the food. Other mechanisms, such as age at first reproduction and embryonic developmental time, also contribute to the competitive outcomes among zooplankton species^29^.

Corresponding to the increased *Microcystis* proportion, different population responses to *Microcystis* were observed: faster to reach the carrying capacity with the subsequent increased population growth rate in *D*. *similoides*, but the opposite case in *M*. *micrura*. This result enriches the species-specific responses in zooplankton population growth to cyanobacteria^30^. Exposure to *Microcystis* would promote the large zooplankton (e.g., *D*. *magna*) to reach its maturity earlier, with the shortened reproduction age^23^. At a population level, these changes in life history traits facilitate the large species reaching its carrying capacity faster. Nonetheless, the biomasses and carrying capacities of the two cladocerans were finally decreased by *Microcystis* treatment (Figs 1, 2 and 4). This is highly related to the nutritional deficiencies and the toxicity of microcystins of the cyanobacteria for the zooplankton^7, 31, 32^. Given the biomass inhibition by *Micorcystis* treatment, the biomass difference between the two cladocerans in competition was enlarged with increased *Microcystis* proportion, as indicated by the increasing *Daphnia*: *Moina* biomass ratio (Fig. 3). It is concluded that the competitive advantage of *D*. *similoides* over *M*. *micrura* was strengthened by *Microcystis* treatment.

Because of the relatively larger gape size with higher filtration on the filamentous cyanobacteria, large *Daphnia* is generally assumed to be more vulnerable to *Microcystis* than small species^33, 34^. Nonetheless, the present strain of *M*. *aeruginosa* grows as unicell in laboratory, and the *D*. *similoides* and *M*. *micrura* are supposed to graze both the toxic and non-toxic cells equally due to their non-selective filtration. *M*. *micrura* assimilated little *Microcystis* when fed only *Microcystis* or even a mixture of *Microcystis* and *Chlorella* ^35^. Given the present low proportions (<35%) of *Microcystis* in diet, large *Daphnia* can minimise the negative influence from cyanobacteria via microcystins detoxification^36^. Zhang *et al*.^37^ studied that the large *D*. *similoides* assimilates low abundance of *Microcystis* with improved reproduction. Using a combined stable-isotope and fatty-acid approach, de Kluijver *et al*.^38^ found that *D*. *similis* consumes live *Microcystis* cells. This supplies additional material and energy for the *D*. *similoides* growth in comparison with *M*. *micrura*. Repeated toxic cyanobacteria- exposure can also increase the tolerance of large *Daphnia* population to toxic *Microcystis* via improving antioxidant systems^39, 40^. In addition, the low nutritional value of cyanobacteria for *Daphnia* promotes the offspring tolerance to toxic *Microcystis* ^41^. Gustafsson *et al*.^13^ studied that previous-exposure to toxic *Microcystis* increased the offspring fitness in *Microcystis* environment. This adaptation may result from improved survival, enhanced reproduction or faster development of offspring^42, 43^. Although some small cladocerans are also studied to develop adaptation^34^, the severer biomass inhibition by *Microcystis* in *M*. *micrura* indicated its weaker adaptation compared with that in *D*. *similoides* in the current study. A comparative study on the phenotypic adaptations between the two species based on individual performances will be performed in the next work. In the presence of *M*. *micrura*, we surprisingly observed that the biomass of *D*. *similoides* at 35% *Microcystis* was slightly higher than that at 20% *Microcystis*. As the *Moina* was severer inhibited by competition with increased *Microcystis*, it is presumed that *Daphnia* in the 35% *Microcystis*-treated cocultures consumed more good food, thereby leading to the relatively higher biomass.

There is a great variation in the influence of cyanobacteria on zooplankton competition. The present observation is not consistent with the general recognition that *Microcystis* promotes the dominance of small-sized cladocerans, but instead supports several investigations demonstrating that large-sized cladocerans are superior competitors in cyanobacteria environment^22, 24, 44^. Besides the concentration tested in present study, many bloom-related variables, such as morphology and toxic property, affect the zooplankton shifts^7, 45–47^. In natural systems, the zooplankton composition during bloom is also regulated by other factors such as temperature and planktivorous fish^48, 49^. Under the background of global warming, increasing temperature would enhance the effects of cyanobacteria on zooplankton with expansive blooms^50–52^. For example, the cladocenran offspring tolerance to toxic *Microcystis* can be promoted by maternal warming^53^. Fish predation also drives the zooplankton fluctuation^54^. The planktivorous fishes affect zooplankton via directly predation or by reducing edible phytoplankton abundance to zooplankton^55^. Recent study showed that planktivorous fishes associated with cyanobacteria promote the zooplankton community shift towards species with good escape ability and *r*-strategy in survival^56, 57^. Therefore, besides the exploration on bloom-relevant factors influencing zooplanktons alone or in combinations, the trophic interactions between planktivorous fish, zooplankton and cyanobacteria require deeper studies to assess the zooplankton community structures during the expansive blooms under warming climate.

## Materials and Methods

### Cladocerans and algal food

Both the *Daphnia similoides* and *Moina micrura* were collected from Taihu Lake in China. The animals were then cultivated in laboratory by feeding 100% *Chlorella pyrenoidosa* at 25 °C for about three years. *C*. *pyrenoidosa* was pre-cultured in liquid BG-11 medium at 25 °C and illuminated at 45 μmol m^−2^ s^−1^ provided by fluorescent lamps in a light–dark period of 14:10 h. Log-phase *C*. *pyrenoidosa* were harvested by centrifugation at 6300 × g for 10 min and used as food. *M*. *aeruginosa* PCC7806 was obtained from the Freshwater Algae Culture Collection of the Institute of Hydrobiology (Wuhan, China). The cyanobacteria produce at least two types of microcystion (MC-LR and MC-RR) with a total content of 3.6 pg per cell via the high-performance liquid chromatography detection^58^. The cyanobacteria were axenically cultured under the same above conditions.

### Experimental protocol

Three food compositions were tested as 100% *C*. *pyrenoidosa* + 0% *M*. *aeruginosa*, 80% *C*. *pyrenoidosa* + 20% *M*. *aeruginosa*, and 65% *C*. *pyrenoidosa* + 35% *M*. *aeruginosa*, with the total carbon content of 1 mg L^−1^. The two cladocerans all died in several days when fed on 50% *Microcystis* in diet based on our pre-experiment. The present-used proportions of *M*. *aeruginosa* were not highly toxic to cause elimination of either *M*. *micrura* or *D*. *similoides*, and thus suitable for competition experiments. Within each food composition, three cladoceran-treatments were set up: (1) 5 *D*. *similoides* (Ds) cultivated alone; (2) 5 *M*. *micrura* (Mm) cultivated alone; (3) 5 Ds and 5 Mm cultivated together. The initial sizes for *D*. *similoides* and *M*. *micrura* were averaged 0.65 mm and 1.2 mm in length on account of a Nis-elements image analyzer coupled with a Nikon light microscope. The experiment was carried out in 1-L beakers containing 500 mL culture media with one specific food composition. The experiment was performed in triplicate, resulting in 3 (food composition) × 3 (cultivation pattern) × 3 (replicate) = 27 beakers. All beakers were maintained at 25 °C in a temperature-controlled chamber and illuminated by 45 μmol m^−2^ s^−1^ fluorescent light with a light–dark period of 14:10 h. To maintain constant food concentrations, we replaced 50% of the medium in each beaker daily with fresh medium with appropriate food abundance. The dry biomass of the animals was estimated via measuring the body length on the basis of the regression curves by Culver *et al*.^59^. The experiment was not terminated until no remarkable increase was detected in the population abundance. When the experiment was finished, the cladoceran populations were composed of individuals in different ages with a length range of 0.7–1.6 mm for *D*. *similoides* and 0.4–0.9 mm for *M*. *micrura*.

The cladoceran biomass versus time was fitted by using the Logistic model:

$${B}_{t}=\frac{K}{1+\frac{K-{{\rm{B}}}_{0}}{{{\rm{B}}}_{0}}{e}^{-rt}},$$

where B_0_ and B_t_ represent the cladoceran biomasses at initial time (t_0_) and time t, *r* represents the population growth rate, and *K* represents the environmental carrying capacity. The biomass inhibition of species in competition was calculated as: biomass inhibition rate (%) = [(biomass in monocultures) − (biomass in cocultures)]/biomass in monocultures × 100%. The biomass ratio of the two cladocerans in cocultures was defined as the biomass of *Daphnia* relative to that of *Moina* (*Daphnia*: *Moina*).

### Statistical analysis

All data are presented as mean ± 1 SE. Two-way ANOVA was used to compare differences between groups in terms of population growth rate, maximum biomass, time to maximum biomass, and the carrying capacity with food combination and absence/presence of competitor as the fixed factors. Significant analyses were followed by Tukey’s post-hoc tests to locate meaningful differences. Statistical analysis was performed using Sigmaplot 11.0 software.
